# 

**Published:** 1904-06-15

**Authors:** 


### ANNOUNCEMENTS.

Address of the Committee on the Promotion of the Appointment of Dentist to the Armies and Navies of the World.—Since the importance of the subject entrusted to the above-named Committee has been very fully appreciated in the United States for the past five years, especially by most of those who make up the accompanying list of American members, it seems unnecessary to more than call your attention to the broader scope of the work involved in an attempt to extend the benefit of an educated and efficient dental service to the armies and navies of the world.

You are invited to contribute papers on the subject and to suggest to the Chairman matter for the report which will pe presented through the full Committee to the *Fourth International Dental Congress at St. Louis.* Please notify the Chairman *at once* what your contribution will be entitled.

It is a matter of regret that the personnel of the Foreign Committee can not now be announced, but is hoped there will yet be such affiliation and co-operative effort as will insure more consideration of the cause we represent by the military and naval authorities of the greater countries of the world. Happily for Americans, the army's and navy's need of the service of dental surgeons is everywhere conceded and only the question of their official status and of the number required remain undetermined. The unsupplied requisitions of Army Department Commanders and Post Surgeons for the service of more dental surgeons not only proves the present corps inadequate, but it proves the *value* of the service more convincingly than the often heard informal expressions of appreciation by army men. Two years ago the Surgeon-General recommended an increase and commissioned rank of permanent tenure. The present Surgeon-General has only recently encouraged the Committee of the National Dental Association to expect his official endorsement of our contention for commissioned rank for military dental surgeons. The attitude of a majority of the Senate and House Military Committees has always been favorable to the profession's contention for commissioned rank.

The different conditions obtaining in the navy, together with the experience in its effort to secure the carrying out of a policy leading to commissioned rank, which policy was adopted before the law providing for the Army Dental Corps was enacted, has warranted the National Dental Association Committee in consistently contending for a status from the start that would insure to the navy dental surgeon the professional, social and official advantages which belong only to commissioned officers.

Friends of the profession in Congress could be depended upon to see that proposed measures providing less than rank should not be adopted, but there was no assurance that rank would be provided until the Navy Department assented thereto. Finally, the Navy Department recommended, in a letter dated March 2, 1904, the appointment by the President of thirty dental surgeons, with rank and pay of Acting Assistant Surgeons and the status of Assistant Surgeons. Assistant Surgeons have a permanent tenure; Acting Assistant Surgeons are removable at the pleasure of the President. This proposal of the Navy Department has been accepted and recommended to the Naval Committees by the authorized representatives of the dental profession. Its enactment is confidently expected by the Congress next winter.

WMS. DONNALLY, *Chairman*,

1018 14th Street, Washington, D. C.

GORDON WHITE, *Asso. Chairman*,

610 Church Street, Nashville, Tenn.

A. J. BROWN, *Secretary*,

1326 L Street, Washington, D. C.

CENTRAL MICHIGAN DENTAL ASSOCIATION.—The Central Michigan Dental Association held its annual meeting at Grand Rapids, April 12, 13, 1904, and elected the following officers: President, C. E. Whitmore, Portland; Vice-president, J. A. Lyon, Grand Ledge; Secretary and Treasurer S. A. Horning, Portland.

Chicago Dental Society.—The annual meeting of the Chicago Dental Society was held April 4th, 1904, and the following officers were elected: President, T. L. Gilmer; 1st Vice-president, C. N. Thompson; 2d Vice-president, F. V. Yorker; Recording Secretary, Winthrop Girling; Corresponding Secretary, A. E. Morey; Treasurer, C. P. Pruyn; Librarian, J. H. Woolley.

—✦—

Ohio Board of Dental Examiners.—The Board of Dental Examiners of the State of Ohio will meet in Columbus June 28–30, 1904, at the Hotel Hartland, for examination of candidates for certificates or registration. Applications should be filed with the Secretary by June 18th. For further particulars address,

H. C. Brown, *Secretary*,
185 E. State St., Columbus.

—✦—

Eastern Indiana Dental Association.—The Eastern Indiana Dental Association held its annual meeting May 4th and 5th, and adjourned to meet next year at Greenfield. The officers chosen are: President, B. S. Binford, Greenfield; Vice-president, R. I. Bell, Greenfield; Secretary and Treasurer, G. E. Stevenson, Liberty.

—✦—

The Canadian Dental Association will hold its next meeting at Toronto, September 6th, 7th and 8th. Visitors from the States will be most cordially welcomed.

W. Cecil Trotter, *Secretary*.

—✦—

The Michigan State Board of Examiners, at its recent meeting in Grand Rapids, examined nearly fifty candidates; less than one-half passed the examinations. Dr. C. H. Oakman, of Detroit, is the new member of the Board.

He was appointed by the Governor recently to succeed Dr. Dennis, of Port Huron, whose term of office had expired. Dr. Oakman has many qualities which will make him valuable in this place.

—✦—

International Congress.—The meetings will be held in the Coliseum and Music Hall building at Thirteenth and Olive Streets, which is admirably adapted for the purpose. This immense building is in the down-town district near all the best hotels and two blocks from the Jefferson Hotel, which is the official headquarters for the Congress officers and members.

The Music Hall, with a seating capacity of 8,000, will be used for the general sessions. Ten section rooms will be provided, so that all sections will have ample space for their sessions.

The building will be equipped with an information bureau, postoffice, cable and telegraph offices, lunch facilities, etc., for the convenience of the members.

The exhibits will be on a large scale, and a feature of the Congress, under the supervision of Dr. Don. M. Gallie, No. 100 State Street, Chicago, to whom application for space should be made at once. The clinics will be in the east nave of the building, under the supervision of J. P. Gray, of Nashville, Chairman of the Committee on Clinics, and his assistants, J. P. Root, of Kansas City, Howard J. McFadden, of Philadelphia, T. P. Hinman, of Atlanta, and other assistants. The clinics promise to be of unusual number and interest. Only members of the Congress will be admitted to the clinics, section or general sessions.

In the Coliseum proper will be held a mammoth banquet on the evening of September 1st, at which the profession's orators of the world will speak. The arrangements of the banquet are in charge of Drs. Geo. A. Bowman, A. H. Fuller, Edw. H. Angle, Adam Flickinger and D. O. M. Le Cron, who have engaged Chas. R. Schrap, the caterer, to serve the banquet.

Other entertainments will be provided the members, their wives and guests, by the local committee, who are considering a reception, trolley or steamboat ride, etc.

On Wednesday evening, August 31st, the twentieth annual meeting of the Supreme Charter of the Delta Sigma Delta Fraternity will be held.

The Committee of Arrangements consists of George Edwin Hunt, chairman, 131 East Ohio Street, Indianapolis, Ind.; D. C. Bacon, Chicago; R. H. D. Swing, Philadelphia; J. H. Kennerly, St. Louis; John Q. Byram, Indianapolis, Ind. They promise an entertainment worthy of the occasion.

All ethical practitioners, whether they are members of any local or State Society, are eligible to membership in the Congress, which must be secured by sending the fee, ten dollars, to the chairman of the Committee on State and Local Organization of the State in which the applicant resides. In return, the chairman sends the member a receipt for the fee, which he forwards to the chairman of the Finance Committee, who sends the member the official receipt, which the member must present to the chairman of the Committee on Registration, who will register all members' names, degrees, address and society affiliations and furnish the member with the card of admission, official badge and programme. The membership fee of ten dollars entitles members to admittance to all sessions and to a copy of the official proceedings of the Congress. None but members will be admitted to the meetings or entitled to take part in the social entertainment.—*Western Journal.*

—✦—

THE Fifth Edition of "The Irregularities of the Teeth," by Eugene S. Talbot, has just come from the press of the S. S. White Dental Manufacturing Company. This edition is practically a reprint of the fourth edition, by a new publishing concern. The work has a value that is too often overlooked by that class of practitioners who study their cases of regulating no farther than to be able to devise the quickest method of accomplishing results. And the criticisms that former editions of this work have met have emanated from those practitioners who do not care to go back of conditions as they find them. While it is true that most cases of irregularity of the teeth have only accidental causes, a considerable portion, no doubt, can be traced to embryological perversions, and doubtless hereditary influences are largely accountable for them. The author's idea that they are atoristic retroversions is difficult of proof, and while this may not be sufficiently demonstrated, the work is a contribution of great value, and represents an enormous amount of study. As such it will be of immense value to future investigators. The work may never come to any considerable use as a text-book, because it does not largely deal with practical regulating of teeth, it is nevertheless a most interesting and valuable book for the student and practitioner. It is published in good form by the S. S. White Dental Manufacturing Company.

